# The Challenge of Antimicrobial Resistance in the Indian Healthcare System

**DOI:** 10.7759/cureus.42231

**Published:** 2023-07-21

**Authors:** Abhishek Sharma, Niketa Thakur, Abhishake Thakur, Ankit Chauhan, Harpreet Babrah

**Affiliations:** 1 Anaesthesia, Sri Guru Ram Das Institute of Medical Sciences & Research, Amritsar, IND; 2 Radiation Oncology, Government Medical College and Hospital, Amritsar, IND; 3 Anaesthesia, Shree Balaji Hospital, Kangra, IND; 4 Cardiac Anaesthesia, UN Mehta Institute of Cardiology and Research Centre, Ahmedabad, IND

**Keywords:** indian healthcare system, antibiotic resistance, overuse and misuse of antibiotics, emerging antibiotic resistance, antibiotic stewardship program, antibiotic policies and guidelines, antimicrobial resistance

## Abstract

The emergence and rapid spread of antimicrobial resistance (AMR) pose a grave threat to public health globally, and particularly so in India. With its unique combination of a dense population, a significant disease burden, and diverse healthcare practices, India stands at a critical juncture in the global battle against AMR. The implications of this escalating crisis are far-reaching, threatening decades of medical progress, undermining healthcare delivery, and posing potential roadblocks to the realization of several Sustainable Development Goals.

AMR is a crisis within the Indian healthcare system as it severely hampers the effective treatment of infectious diseases, leading to higher mortality rates, longer hospital stays, and increased healthcare costs. Its rise could mark the return of the pre-antibiotic era, where common infections and minor surgeries could once again become life-threatening. Addressing the challenge of AMR in India requires a comprehensive, multifaceted, and well-coordinated response. From creating strong regulatory frameworks for antibiotic usage and improving diagnostic capabilities to fostering greater public awareness and promoting research into new antimicrobials, the strategies need to be as diverse and interconnected as the problem itself.

This editorial will delve into the specificities of the AMR challenge within the Indian healthcare system, discuss potential strategies for mitigating the crisis, and evaluate the broader implications for public health and national policy. It will also highlight why India's response to this global health threat is crucial not only for the country but for the world at large.

## Editorial

The phenomenon of antimicrobial resistance (AMR) refers to the ability of microorganisms, such as bacteria, viruses, fungi, and parasites, to evolve and become resistant to the drugs that were previously effective against them. This resistance primarily stems from the misuse and overuse of antimicrobials in humans, animals, and even in the environment. The Indian Council of Medical Research (ICMR) annual report of 2021 found that *Klebsiella pneumoniae*'s susceptibility to imipenem has decreased from 65% in 2016 to 43% in 2021, while *Escherichia coli*'s susceptibility to imipenem has decreased from 86% in 2016 to 64% in 2021 [1]. This ICMR report also found that isolates of *Escherichia coli* and *Klebsiella pneumoniae* that are resistant to carbapenems were also resistant to other antimicrobials, making it difficult to treat carbapenem-resistant infections. In addition, they found that in 87.5% of study participants, *Acinetobacter baumannii* infections were resistant to the broad-spectrum antimicrobial carbapenem, reducing the number of available treatment choices. Similarly, a study by Sharma et al. found that the sensitivity of *Klebsiella pneumoniae* to meropenem decreased from 15% in 2018 to 2.5% in 2022, while the sensitivity of *Klebsiella* to colistin decreased from 96% in 2018 to 28% in 2022 [2]. This AMR situation continues to escalate in India due to several key issues [3,4]. We have summarized the potential factors that contribute to the rising AMR in India in Table 1.

**Table 1:** Contributing factors responsible for worsening antimicrobial resistance in India

Sr. No.	Factors responsible for worsening antimicrobial resistance (AMR) in India	
1	Overuse and misuse of antibiotics	One of the most significant drivers of AMR is the misuse and overuse of antibiotics. In India, antibiotics can often be obtained without a prescription, leading to their widespread and often unnecessary use.
2	Lack of awareness	Public understanding of AMR remains low in India. Many people do not understand the implications of not finishing a course of antibiotics or using them without necessity, which can lead to bacteria evolving to withstand these medications.
3	Poor infection control	Infection control measures in healthcare facilities can be suboptimal in India, leading to high rates of hospital-acquired infections, which often involve drug-resistant organisms.
4	Lack of regulation in animal husbandry	Antibiotics are commonly used in animal farming as growth promoters and to prevent disease. However, this can lead to antibiotic residues in meat and other animal products, contributing to antibiotic resistance in humans.
5	Limited access to clean water and sanitation	This leads to a higher prevalence of infectious diseases, which in turn can drive higher usage of antibiotics and contribute to the development of AMR.
6	Inadequate surveillance and regulation	India lacks a robust surveillance system for monitoring AMR, making it difficult to grasp the full extent of the problem and design appropriate interventions. Regulations on antibiotic use are also insufficiently enforced.
7	Challenges in the Indian pharmaceutical industry	India is a major producer and consumer of antibiotics, and there have been concerns about the lack of regulatory oversight of antibiotic production and disposal, which could contribute to environmental contamination and the spread of AMR.

While AMR is a widespread issue across India, the patterns and causes of resistance can differ between rural and urban areas due to various socio-economic, environmental, and healthcare-related factors [5]. In urban areas, overpopulation can contribute to the rapid spread of infections. Furthermore, urban environments may have more pollution, including pollution from unregulated pharmaceutical waste disposal, which can also contribute to AMR.

In contrast, rural areas may have different drivers of AMR. While there may be less access to antibiotics in general due to the lower availability of healthcare services, misuse can still occur due to a lack of awareness about the correct use of antibiotics. Over-the-counter sales of antibiotics without a prescription, a common practice in India, may be particularly widespread in rural areas where healthcare access is limited. Furthermore, the use of antibiotics in agriculture, including in livestock, can be a significant driver of AMR in rural areas. Rural-urban migration can also contribute to the spread of AMR between these areas. People moving from rural areas to cities can bring resistant bacteria with them, while people returning to rural areas after receiving medical treatment in cities can introduce resistant bacteria into these rural settings.

AMR patterns can vary between the private and public healthcare systems in India due to differences in practices, policies, and resources available in each sector [6]. In private healthcare settings, there is a tendency to over-prescribe antibiotics, either to meet patient expectations or as a prophylactic measure. This over-prescription can contribute to the development of AMR. The private healthcare sector may have access to a wider range of antibiotics, including newer and more potent ones. While this can be beneficial in treating infections, it can also contribute to the emergence of resistant strains if these antibiotics are not used judiciously. The private healthcare sector often caters to a more affluent patient population, which might have different patterns of antibiotic usage compared to the general population.

On the other hand, the public healthcare system often faces resource constraints, which can affect the availability and usage patterns of antibiotics. In some cases, this can lead to the inappropriate use of antibiotics due to the unavailability of the most appropriate ones. Public hospitals usually have a higher patient load, and the sheer number of patients might sometimes result in less personalized care. This could lead to a lack of follow-up on antibiotic courses and potential misuse. In public hospitals, especially in lower-tier cities and rural areas, infection control practices may not be as robust as in private hospitals. This can lead to the spread of drug-resistant infections within the healthcare facility. Patients in public hospitals often come from lower socio-economic backgrounds, and their previous exposures to antibiotics (often without prescription) can impact the AMR patterns seen in these settings.

The future of AMR in India, as in other parts of the world, will depend heavily on the measures taken to address this issue at the individual, community, national, and international levels [7]. To tackle the global challenge of AMR, the World Health Organization (WHO) and the ICMR have jointly introduced several initiatives [8,9]. These initiatives have a primary emphasis on advancing surveillance methods, raising awareness, conducting research, and facilitating the development of new antimicrobial treatments. Table 2 provides a summary of a complete strategy for dealing with the threat of AMR.

**Table 2:** Strategies to reduce antimicrobial resistance in the Indian healthcare system

A comprehensive strategy for addressing the problem of antimicrobial resistance (AMR) may include:	
1	Strengthening regulatory oversight of antibiotic use in humans and animals, and of antibiotic production and disposal.
2	Increasing investment in healthcare infrastructure, including diagnostic facilities.
3	Implementing public education campaigns to raise awareness of AMR and promote the appropriate use of antibiotics.
4	Improving surveillance of AMR in both private and public healthcare systems to understand and address the issue better.
5	Fostering collaboration between the private and public sectors to share best practices and resources for combating AMR.
6	Urban strategies may need to focus more on reducing pharmaceutical pollution and the over-prescription of antibiotics.
7	Rural strategies may need to focus on improving access to quality healthcare and education about appropriate antibiotic use.
8	Promoting research and development of new antibiotics and alternative treatments for bacterial infections.
9	Collaborating with international partners to share best practices and coordinate efforts to tackle AMR.
